# Integrating Shared Decision-Making into Undergraduate Oncology Education: A Pedagogical Framework

**DOI:** 10.1007/s13187-024-02419-8

**Published:** 2024-03-06

**Authors:** Aaron Lawson McLean, Anna C. Lawson McLean

**Affiliations:** Department of Neurosurgery, Jena University Hospital – Friedrich Schiller University Jena, Am Klinikum 1, 07747 Jena, Germany

**Keywords:** Oncology education, Shared decision-making, Undergraduate medical curriculum, Patient-clinician collaboration, Evidence-based medicine, Medical pedagogy

## Abstract

The integration of shared decision-making (SDM) into undergraduate oncology education represents a critical evolution in medical pedagogy, reflecting the growing complexity and patient-centric focus of contemporary healthcare. This paper introduces a comprehensive pedagogical framework designed to embed SDM within the undergraduate medical curriculum, particularly in oncology, where the multiplicity of treatment options and their profound impact on patient life underscore the necessity of this approach. Grounded in a systematic literature review and aligned with established educational theories, this framework proposes twelve strategic approaches to cultivate future physicians proficient in both clinical acumen and patient-collaborative decision-making. The framework emphasizes real-world clinical experience, role-playing, case studies, and decision aids to deepen students’ understanding of SDM. It advocates for the development of communication skills, ethical deliberation, and cultural competence, recognizing the multifaceted nature of patient care. The inclusion of patient narratives and evidence-based decision-making further enriches the curriculum, offering a holistic view of patient care. Additionally, the integration of digital tools within the SDM process acknowledges the evolving technological landscape in healthcare. The paper also addresses challenges in implementing this framework, such as curricular constraints and the need for educator training. It underscores the importance of continual evaluation and adaptation of these strategies to the dynamic field of medical education and practice. Overall, this comprehensive approach aims not only to enhance the quality of oncological care but also to prepare medical students for the complexities of modern medicine, where patient involvement in decision-making is both a necessity and an expectation.

**Supplementary Information:**

The online version contains supplementary material available at 10.1007/s13187-024-02419-8.

## Introduction

The concept of shared decision-making (SDM) has undergone a significant evolution, mirroring the shifts in the broader healthcare landscape. Historically, medical decision-making was predominantly clinician-driven, with limited patient involvement. This paradigm shifted notably over the past few decades, as patient autonomy and individual rights gained prominence. The ethos of SDM emerged from this transition, advocating for a more egalitarian approach to healthcare, where patient preferences and values are integrated into the decision-making process [1].

In oncology, this shift is particularly salient. The field has witnessed an exponential growth in therapeutic options, ranging from targeted therapies to immunotherapies, each accompanied by its own risk–benefit profile. This burgeoning complexity makes SDM not only desirable but also essential. SDM in oncology respects the patient’s right to be an active participant in their care, acknowledging the profound personal impact of oncological decisions [2, 3].

Despite the acknowledged importance of SDM, there remains a significant gap in its integration into medical education, particularly at the undergraduate level [4]. Traditional medical curricula often remain focused on the biomedical model, with less emphasis on the skills necessary for effective SDM, such as communication, ethical deliberation, and appreciation of patient values. This educational shortfall is increasingly incongruent with the demands of contemporary oncological practice, where patient involvement in decision-making is not just preferred but expected [5].

This paper, therefore, seeks to address this gap. It proposes a comprehensive pedagogical framework that aligns with the current needs of oncological care. By outlining twelve strategic approaches, it aims to embed the principles and practices of SDM into the undergraduate medical curriculum, ensuring that future physicians are not only clinically adept but also proficient in collaboratively navigating the complex decision-making landscape of modern oncology.

## Methods

### Systematic Literature Review

The literature review was conducted to encompass a broad spectrum of sources relevant to SDM, oncological care, and medical education. The selection criteria for literature included peer-reviewed articles published within the last 15 years, ensuring contemporary relevance. We focused on studies that discussed the implementation of SDM in clinical practice, its integration into medical curricula, and the impact of SDM on patient outcomes in oncology. Databases including PubMed, EMBASE, CINAHL, PsychINFO, and Web of Science were comprehensively searched. Additionally, reference lists of pertinent articles were scrutinized to identify additional relevant literature. The inclusion criteria were stringent, favoring studies that provided empirical evidence or substantial theoretical frameworks regarding SDM in medical education and oncological practice. For a detailed exposition of the search strategy employed, including the specific adaptations made to accommodate the unique indexing and search functionalities of each database, readers are directed to Supplementary information 1.

### Pedagogical Alignment

Upon identifying strategies from the literature review, the next step involved aligning these strategies with established pedagogical principles. This alignment ensured that the proposed SDM teaching methods were not only evidence-based but also educationally sound and coherent with broader educational objectives. Key pedagogical frameworks considered included experiential learning, reflective practice, adult learning theory, and competency-based education. Each identified SDM strategy was scrutinized and adapted to align with these pedagogical principles, ensuring that they could be seamlessly integrated into existing medical curricula and effectively facilitate the learning objectives of SDM.

This dual approach of rigorous literature review and pedagogical alignment culminated in the development of a comprehensive, evidence-based, and pedagogically sound framework for incorporating SDM into undergraduate medical education, particularly in the field of oncology.

## Results: Identified Strategies

### Strategy 1: Ground the Concept of SDM in Real-World Oncology Scenarios

To foster a profound understanding of SDM in oncology, it is vital for students to be immersed in clinical settings where SDM is actively practiced [1]. Observing patient consultations offers a practical framework for learning [6]. In these settings, students can observe firsthand how diagnoses, treatment options, and potential outcomes are communicated, aligning clinical evidence with individual patient preferences. In the realm of oncology, where decisions carry significant weight regarding patient outcomes and quality of life, such practical experiences serve to solidify the foundational principles of SDM [7]. It is through this hands-on exposure that students can critically analyze and understand the complexities and importance of integrating clinical evidence with patient values in the decision-making process [8].

### Strategy 2: Facilitate Role-Playing Exercises

Role-playing remains an effective pedagogical method, especially in contexts that involve interpersonal communication and decision-making [9–11]. To deepen comprehension of SDM in oncology, students can engage in simulated patient-provider consultations through role-playing. By alternating roles in these simulations, students gain insights into the challenges faced by healthcare providers and patients alike. These exercises foster effective communication, especially in conveying intricate treatment options and their implications [12]. Additionally, they offer a platform for students to cultivate empathy, a critical skill for addressing sensitive discussions inherent to oncology [13, 14]. Through repeated practice in controlled settings, students can better prepare for real-world oncology consultations, ensuring a balanced integration of patient values and clinical evidence in medical decisions.

### Strategy 3: Dissect Case Studies with Varied Outcomes

Case studies serve as invaluable tools in medical education, offering tangible examples that bridge theory with practice [15]. In exploring the complexities of SDM in oncology, it might be beneficial to expose students to diverse case studies, each demonstrating varied outcomes of the decision-making process. Such studies, especially those highlighting the divergence between patient values and standard medical recommendations, offer insight into the layered nature of SDM. Within oncology, where treatment choices influence both life quality and duration, an appreciation for the range of outcomes influenced by SDM becomes evident [16]. Through examination of these cases, students may gain a deeper understanding of the role of patient values and their potential interplay with clinical advice [17]. This approach aims to prompt students to consider the weight of patient values and how they interplay with clinical recommendations, preparing them for the nuanced discussions they will encounter in their professional practice.

### Strategy 4: Introduce Decision Aids

In the innately complex domain of oncology, the decision-making process is often augmented by a suite of decision aids designed to facilitate understanding and discussion [18, 19]. Medical educators should introduce students to these tools, commonly found in the form of risk diagrams, outcome probability charts, and decision trees [20]. These aids aim to make abstract concepts more tangible, allowing patients to visualize potential outcomes, benefits, and risks associated with each treatment option [21]. Training sessions could be designed wherein students learn to employ these aids not merely as passive reference tools but as active instruments for dialog, ensuring that patients comprehend the vast array of information presented to them [22]. By familiarizing themselves with these decision aids, students can foster transparent and productive discussions with patients, anchoring the SDM process in both evidence-based data and individual patient perspectives.

### Strategy 5: Emphasize the Ethics of SDM in Oncology

Oncological care is replete with ethical challenges that arise when aligning medical recommendations with patient preferences [23–25]. It is crucial for undergraduate medical students to understand these ethical dimensions to navigate potential dilemmas in SDM. As educators, it is essential to guide students in recognizing the nuances between providing hope, setting realistic expectations, and honoring patient autonomy [26]. Incorporating discussions that delve into scenarios where patient desires might diverge from standard medical guidelines can prove insightful [27, 28]. By analyzing these situations, students can explore methods to approach discrepancies between clinical evidence and patient wishes. Ethical considerations also extend to matters of informed consent, treatment discontinuation, and end-of-life choices [29]. Through structured discussions and case analyses, students can be trained to handle these situations with integrity, ensuring decisions are respectful of both medical guidelines and patient values.

### Strategy 6: Teach Communication Skills Specific to Oncology

Effective communication is paramount in oncology, where conversations often involve sensitive topics like prognosis and end-of-life care [30, 31]. Medical students must be proficient in tailored verbal and non-verbal communication techniques for such scenarios [32]. Developing focused training modules can enhance skills in delivering clear information while demonstrating empathy. Students should practice conveying complex medical terminology in understandable terms, ensuring comprehensive comprehension by patients and their families. Proficiency in interpreting non-verbal cues and responding appropriately is essential. Role-play exercises can be valuable in this context, enabling students to simulate real consultations [33]. Constructive feedback mechanisms are crucial, aiding students in adapting their communication styles. This rigorous training can equip students with the capacity to establish trust, foster understanding, and collaborate effectively with patients during the SDM process.

### Strategy 7: Advocate for Continual Reflection

The practice of self-reflection is paramount for medical students to critically assess their understanding and application of SDM in oncology [34]. Encouraging students to introspect regularly allows them to identify biases, challenge pre-existing notions, and refine their approach to SDM [35, 36]. Incorporate structured reflective exercises into the curriculum, such as journaling assignments. These should prompt students to reflect upon their experiences in real-world clinical settings, the challenges they faced, and the decision-making processes they observed or participated in [37]. Through this, they can consolidate their learning experiences and analyze them in the context of theoretical SDM principles [38]. Furthermore, fostering reflective group discussions can be beneficial [39, 40]. Such sessions provide a platform for students to share experiences, gain insights from peers, and collectively evolve their perspectives on SDM. This collaborative form of reflection can aid in highlighting the multifaceted nature of oncological care and emphasize the importance of continual learning in the ever-evolving field of oncology.

### Strategy 8: Organize Interdisciplinary Collaborations

Oncology, by its nature, demands a multifaceted approach to care. Interdisciplinary collaborations are critical for providing comprehensive patient care, and they play a pivotal role in SDM [41]. For medical students, understanding the roles and perspectives of various healthcare professionals is crucial for effective SDM [42, 43]. Incorporating structured interdisciplinary sessions into the curriculum where students can interact with professionals such as nurses, pharmacists, social workers, and others involved in oncological can offer students a broader understanding of the SDM process from varied professional standpoints [44]. It allows them to appreciate the contributions of each discipline and how they interplay in the decision-making process [45, 46]. Such interdisciplinary engagements not only provide diverse perspectives but also emphasize the team-based nature of oncological care [47]. By understanding the different facets of a patient’s care team, students are better prepared to engage in collaborative SDM, ensuring that decisions are well-rounded, considerate of various expert opinions, and are in the best interest of the patient.

### Strategy 9: Highlight Cultural and Social Sensitivities

SDM in oncology is not conducted in a cultural vacuum. Cultural, social, and individual backgrounds play a critical role in shaping patients’ preferences, values, and decisions about their care [48, 49]. To enhance the depth of understanding for medical students, it is essential to expose them to diverse patient populations [50, 51]. By interacting with patients from various backgrounds, students gain insights into how sociocultural factors influence treatment choices [52, 53]. This exposure can be facilitated through case studies, clinical rotations in diverse settings, or structured interactions with patients of varied backgrounds [54–56]. Furthermore, sessions on cultural competence may be integrated into the curriculum. These sessions should provide students with knowledge and tools to recognize and respect cultural variations in health beliefs, values, and practices [56, 57]. Emphasizing the importance of cultural competence ensures that students recognize the inherent biases that may arise in clinical interactions, thereby striving for more equitable care. By focusing on the cultural and social sensitivities that impact SDM in oncology, students are better equipped to make decisions that are both medically sound and culturally sensitive, ensuring a more holistic approach to patient care.

### Strategy 10: Integrate Patient Narratives

In oncology, where decisions can have profound implications, understanding the patient’s perspective is paramount [58]. One of the most effective ways to convey this perspective to medical students is through direct narratives from patients or their family members. Inviting patients to share their experiences with diagnosis, treatment, and decision-making offers an invaluable perspective [59]. These personal accounts can illuminate the real-world implications of SDM, highlighting both the challenges faced and the benefits realized when patients are active participants in their care decisions [2, 60]. Furthermore, patient narratives can underscore the emotional dimensions of oncological care, which are often not fully captured in clinical case studies or textbooks [61]. They provide students with a firsthand look at the fears, hopes, and uncertainties that patients grapple with, emphasizing the human aspect of medical practice [62]. To optimize the learning from these sessions, it is advisable to follow up with debriefing sessions [63]. Here, students can discuss their takeaways, clarify doubts, and reflect on how these narratives can influence their future practice. By integrating patient narratives into the curriculum, medical students may gain a deeper appreciation for the central role of the patient in SDM, reinforcing the importance of empathy and active listening in clinical practice [64].

### Strategy 11: Encourage Evidence-Based Decision-Making

Incorporating evidence-based medicine (EBM) into the SDM process is paramount in oncology [65]. While SDM emphasizes patient values and preferences, it is equally crucial for decisions to be grounded in the most recent and relevant evidence. Medical students should be trained to approach clinical situations with a dual lens: one that views the patient’s individual needs and values, and another that scans the existing scientific literature for applicable data [66]. An emphasis should be placed on teaching students the skills to critically evaluate medical research, discerning the quality and relevance of studies [67]. Practical exercises could involve presenting students with a clinical scenario, alongside several research studies, and tasking them with incorporating this evidence into a mock patient consultation. By doing so, they practice weaving together patient values with scientific findings. Moreover, it is important for students to understand that evidence-based recommendations can change over time as new research emerges. Educators should underscore the need for ongoing learning and adaptation in practice, instilling in students a respect for the dynamic nature of medical evidence. This approach not only bolsters the quality of care but also aligns medical interventions closely with patient preferences [68].

### Strategy 12: Embrace Digital Tools for SDM

The digital landscape offers a promising avenue to enhance SDM in oncology [69–71]. Medical students, as digital natives, stand at the precipice of integrating technology seamlessly into clinical practice, and educators should foster this [72, 73]. Students should be introduced to the concept that various digital tools can assist in the SDM process. For instance, there are platforms designed to visually represent medical data, making it more digestible for patients [74–76]. These visualization tools can help bridge the comprehension gap, turning abstract data into meaningful insights, allowing for more informed decisions. Another promising avenue is symptom-tracking applications [77, 78]. While students do not need to be versed in specific apps, they should be aware of the potential such tools offer. Regular digital updates on a patient’s well-being can guide discussions and assist in tailoring personalized treatment plans [79]. It is also paramount for students to understand the ethical considerations surrounding digital tools, especially regarding data security and patient privacy [80]. As they progress in their careers, this foundation will enable them to evaluate and incorporate emerging technologies judiciously into their practice, always keeping the patient’s best interests at the forefront [81].

## Discussion

The strategies presented in this manuscript offer a comprehensive approach to integrating SDM practices into undergraduate medical education, specifically within the context of oncology. While these strategies provide a robust framework, there are certain challenges and considerations that merit discussion to ensure the successful implementation and enhancement of SDM education.

### Challenges and Limitations

The incorporation of SDM into medical education faces several challenges, including constraints on curricular time, competing educational priorities, and potential resistance stemming from entrenched traditional teaching methodologies. These hurdles necessitate a proactive and strategic approach to ensure successful integration. Key to overcoming these obstacles is the development of workshops and training sessions specifically designed for educators. These sessions should aim to not only acquaint educators with the SDM strategies but also to highlight their significance and positive impact on patient care. Furthermore, tailoring these strategies to suit the specific resources and time limitations of each educational institution is critical for enhancing the practicality and effectiveness of their implementation. Recognizing and actively addressing these challenges with pragmatic solutions is essential to create a conducive environment for the seamless integration of SDM into the medical curriculum.

### Cultural and Social Sensitivities

SDM in medical practice is not solely based on medical evidence and individual patient preferences; it also necessitates a keen awareness of cultural and social nuances. The influence of a patient’s cultural background, beliefs, and social milieu significantly shapes medical decision-making [82]. Educational exposure to a diverse array of patient populations through case studies and clinical interactions is vital for medical students to comprehend the impact of these sociocultural factors on treatment decisions. Training in cultural competence is imperative to provide students with the necessary skills to adeptly navigate these multifaceted aspects, thereby promoting respectful and equitable patient-centered care [83]. By integrating cultural and social considerations into the SDM process, medical students are better equipped to participate in meaningful decision-making discussions, effectively acknowledging and respecting individual patient values within the rich tapestry of cultural diversity [43, 84].

### Assessment and Evaluation

The effectiveness of the implemented SDM strategies within the educational framework can be appraised through a variety of evaluative measures. Objective assessment instruments, such as standardized patient encounters, are pivotal for gauging students’ proficiency in applying SDM principles in practical clinical contexts [85]. Additionally, self-assessment methodologies offer students the opportunity for introspection, enabling them to critically assess their progression in comprehending and implementing SDM concepts throughout their educational journey [86]. Furthermore, soliciting feedback from both students and educators regarding the perceived efficacy of these strategies is crucial for the iterative refinement of the educational approach. Continuous evaluation is essential to ensure that the learning objectives are met and that the strategies adaptively align with the dynamic requirements of medical education and evolving patient care paradigms.

### Holistic Approach to Decision-Making

SDM extends beyond basic communication skills, necessitating a comprehensive and integrative approach to medical decision-making. The strategies proposed in this paper advocate for an educational model that transcends traditional clinical evidence and patient preferences, incorporating ethical considerations, interdisciplinary collaboration, patient narratives, and the pragmatic use of digital tools. This amalgamation aims to equip medical students with the capability to proficiently address the diverse and complex aspects of oncological care, including its emotional, ethical, and technological dimensions. By adopting this multifaceted approach, the framework aspires to enhance the overall quality of patient care and to prepare future medical practitioners for the intricate and evolving landscape of contemporary medicine, albeit with an understanding of the inherent challenges and limitations of such an integrative approach.

## Conclusion

In synthesizing the necessity for embedding SDM within undergraduate oncology education, this paper presents a framework that aligns with the evolving dynamics of medical practice, particularly in the realm of oncology. The strategies outlined herein propose a methodological shift in medical education, moving toward a model that balances clinical expertise with patient-centered decision-making.

This framework, derived from a systematic literature review and attuned to established pedagogical theories, recognizes the intricacies of oncological care and the heterogeneity of patient encounters. Strategies such as the incorporation of real-world clinical experiences, simulated patient interactions, analytical case studies, and the use of decision aids are posited as essential components in cultivating a deeper understanding of SDM. Furthermore, the emphasis on communication skills, reflective practice, interdisciplinary collaboration, cultural competence, and digital literacy aims to equip students with a diverse skill set necessary for contemporary oncological practice.

The transition toward a curriculum that emphasizes patient autonomy and collaborative decision-making mirrors the broader shift in healthcare toward more patient-inclusive models. This is particularly relevant in oncology, where treatment options and patient preferences are notably complex. However, the implementation of these strategies requires careful consideration and adaptation to the specific contexts of educational institutions. The proposed framework, while comprehensive, is not without its limitations and should be viewed as a starting point for further development and customization.

Educators and practitioners in the field of medicine carry the responsibility of shaping future medical practice. This framework represents a considered approach to this responsibility, acknowledging the evolving nature of medical science and the importance of patient-physician relationships. However, it must be noted that the integration of these strategies into medical curricula requires ongoing evaluation and adaptation to ensure their effectiveness and relevance.

In summary, the integration of SDM into the undergraduate oncology curriculum is a critical step toward enhancing patient-centered care in medical practice. This manuscript offers a foundational framework for this integration, providing a structured yet adaptable approach. It is imperative that medical educators and institutions approach the adoption of these strategies with a discerning perspective, ensuring that they are effectively integrated into the fabric of medical education and practice.

### Supplementary Information

Below is the link to the electronic supplementary material.

Supplementary File 1 (DOCX 20 KB)

## Data Availability

All data pertinent to this study are encompassed within the article. No supplementary data were generated or analyzed during the creation of this manuscript.
