# Supplementary material for: Integrating Shared Decision-Making into Undergraduate Oncology Education: A Pedagogical Framework
Source: J Cancer Educ. 2024 Mar 6;39(4):374–82. doi: 10.1007/s13187-024-02419-8 (PMC11219368; doi:10.1007/s13187-024-02419-8)
Supplement: Supplementary file 1 — (DOCX 20 KB) [file 13187_2024_2419_MOESM1_ESM.docx]

**Supplementary File 1: Search Strategy**

The following PubMed search was performed on 8th August 2023:

("shared decision making"[Title/Abstract] OR "SDM"[Title/Abstract] OR "patient autonomy"[Title/Abstract] OR "collaborative decision-making"[Title/Abstract] OR (Patient Participation[MeSH Major Topic]) OR ("Health Knowledge, Attitudes, Practice"[MeSH Major Topic]) OR (Patient Satisfaction[MeSH Major Topic]) OR (Decision Making, Shared[MeSH Major Topic]) OR (Patient Education as Topic[MeSH Major Topic]) OR (Physician-Patient Relations[MeSH Major Topic]) OR (Decision Support Techniques[MeSH Major Topic]) OR (Patient Preference[MeSH Major Topic])) AND ("oncolog*"[Title/Abstract] OR "cancer care"[Title/Abstract] OR "tumor care"[Title/Abstract] OR "tumour care"[Title/Abstract] OR (Medical Oncology[MeSH Major Topic]) OR (Neoplasms[MeSH Major Topic])) AND ("medical education”[Title/Abstract] OR "undergraduate medical education"[Title/Abstract] OR "undergraduate education"[Title/Abstract] OR "undergraduate curriculum"[Title/Abstract] OR "medical curriculum"[Title/Abstract] OR "medical training"[Title/Abstract] OR "pedagog*"[Title/Abstract] OR "educational objectives"[Title/Abstract] OR “learning”[Title/Abstract] OR "integration"[Title/Abstract] OR "incorporation"[Title/Abstract] OR "implementation"[Title/Abstract] OR "best practices"[Title/Abstract] OR "teaching strategies"[Title/Abstract] OR "teaching methodolog*"[Title/Abstract])

Timeframe: The operator *("2008"[Date - Completion] : "2023"[Date - Completion])* was used to restrict the timeframe to the last 15 year, to foreground contemporary perspectives

Article language was restricted to English or German.

No other restrictions were applied.

**Adaptation for Other Databases and Dates Searched:**

1. Embase, searched on 9th August 2023 - Search terms were adapted using Emtree terms and free-text searching to match the relevant controlled vocabulary for this database.
2. PsycINFO, searched on 9th August 2023 - The strategy was modified to include terms related to psychology, behavioral sciences, and medical education, using PsycINFO's thesaurus terms.
3. Web of Science, searched on 9th August 2023 - Keywords and Boolean operators were used as in the PubMed search but adapted to meet the database's syntax and controlled vocabulary.
4. CINAHL, searched on 9th August 2023 - The search was adapted using CINAHL Headings and tailored to include terms specific to nursing and allied health education.

These adaptations ensured the search strategy was appropriately broad and thorough, tailored to each database's unique features and subject scope.
